# Surface Defects and Symmetry Breaking Impact on the Photoluminescence of InP Quantum Dots

**DOI:** 10.1021/acs.nanolett.5c02317

**Published:** 2025-06-17

**Authors:** Surender Kumar, Caterina Cocchi, Torben Steenbock

**Affiliations:** † Institut für Festkörpertheorie und -Optik, 9378 Friedrich-Schiller-Universität Jena, 07743 Jena, Germany; ‡ Department of Chemistry, 232751 University of Hamburg, HARBOR, Building 610, Luruper Chaussee 149, Hamburg 22761 Germany; § Institute of Physics and Center for Nanoscale Dynamics (CeNaD), 14915 Carl von Ossietzky Universität Oldenburg, 26129 Oldenburg, Germany

**Keywords:** Indium Phosphide, Photoluminescence, DFT, Exciton Fine Structure, Surface Traps

## Abstract

To fully uncover the potential of indium phosphide (InP) quantum dots (QDs) for optoelectronics, it is crucial to understand how surface defects impact their photoluminescence (PL). To address this question, we investigate the excitonic properties of defective InP QDs using two-component density functional theory and screened configuration interaction singles. In agreement with earlier observations, we identify 3-fold coordinated phosphorus surface atoms, which function as hole traps, as the major contributors to PL. Additionally, we find that electron traps of 3-fold coordinated indium atoms, quenching the band-edge PL, can further contribute to trap PL, if they lie within the single-particle gap. Importantly, our calculations reveal that surface-induced symmetry breaking leads to fundamentally different exciton fine structures in excellent agreement with measurements. This study underscores the significant influence of surface imperfections on InP QD PL and provides a refined framework for interpreting their optical properties.

## Graphical abstract

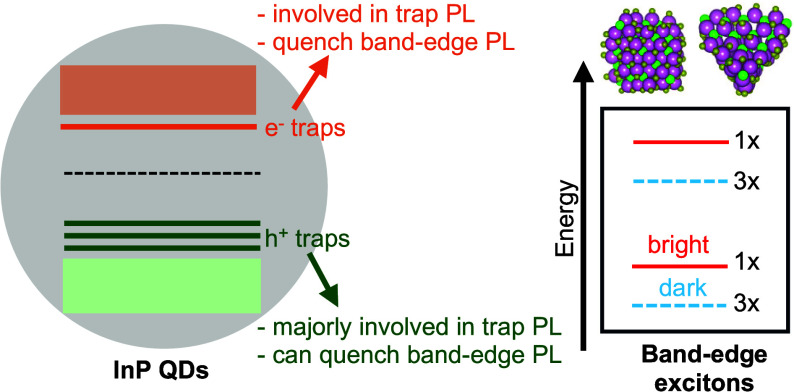

Colloidal indium phosphide (InP) semiconducting quantum dots (QDs) have emerged as a safer alternative to CdSe QDs,^1,2^ possessing similar photoluminescence (PL) properties.^3–5^ Their combined low toxicity and favorable optical properties make InP QDs very attractive for several applications, including displays,^6^ light-emitting diodes,^7–10^ and optoelectronic devices.^5,11,12^ All the above-mentioned technologies demand sharp PL features and high quantum yields. Experimental studies have showed that these requirements are difficult to meet in core-only InP QDs due to the strong influence of surface defects on the PL properties.^13–20^ First, surface defects enhance the nonradiative decay to the ground state, which results in quantum yields below 1% for core-only QDs.^16,17,21^ Second, surface defects are known to form emissive trap states below the band edges, contributing to the rather broad emission of InP QDs.^3,15,17^

To improve the PL properties of these systems, different strategies have been devised to remove surface defects, including the growth of heteroshells around the InP core,^3,22^ HF edging,^20,23,24^ and Z-type ligand passivation.^13,16,25,26^ Although, these attempts contributed to improve the PL properties of InP QDs, none of them was able to completely remove surface defects. Spectroscopic studies revealed the role of hole and electron traps in the trap PL of InP QDs,^15,17,19^ suggesting that hole traps are primarily involved in trap PL, while electron traps quench band-edge PL. Density functional theory (DFT) calculations subsequently showed that hole (electron) traps originate mainly from 3-fold coordinated P (In) atoms at the QD surface.^19,27,28^ While these studies offered valuable insights into the energies of trap states, they could not explain the nature of the excitonic states involved in PL.

Additional open questions concern the band-edge PL, which has been the subject of several studies in the literature.^17,22,29^ Temperature-dependent PL measurements confirmed the existence of a dark excitonic ground state and a higher-lying bright state, which becomes thermally occupied with increasing temperature.^17,22^ To explain the temperature-dependent PL properties, the excitonic fine structure,^30^ namely, the arrangement and brightness of the band-edge exciton, is of crucial importance. The early study by Franceschetti et al.,^29^ obtained for spherical zincblende InP QDs in the framework of screened configuration interaction singles (SCIS) calculations, represents a commonly adopted reference for the excitonic fine structure of InP QDs. In that work, the QDs were assumed with *T*_*d*_ symmetry, leading to a significant symmetric excitonic fine structure. Although such high symmetries are commonly assumed in theoretical studies on colloidal semiconducting QDs,^30–36^ they reproduce idealized structures whereby the influence of the QD surface is ignored. In our previous studies on CdSe QDs,^37–39^ we showed that the coordination of ligands to the QD surface and surface defects considerably break the symmetry of the system, leading to fundamentally different excitonic fine structures compared with those of highly symmetric models.

In this work, we study the symmetry breaking in the excitonic fine structures of defective InP QDs using hybrid DFT and SCIS calculations. We identify the roles of the 3-fold coordinated phosphorus and indium surface atoms as hole and electron traps, respectively, elucidating their contributions to trap-state emission and quenching of band-edge luminescence. We demonstrate that the inherent asymmetry introduced by surface defects and the ligand environment leads to a distinct 3–1–3–1 splitting in the band-edge exciton fine structure, contrasting with predictions for idealized, highly symmetric quantum dots. The validity of our findings is supported by the excellent agreement between our calculations and experimental PL measurements, highlighting the necessity of considering realistic surface structures for accurately predicting the optical properties of InP QDs.

In this analysis, we consider five different F-passivated, quasi-spherical zincblende InP QDs with diameters of approximately 2 nm, as considered in a previous study^28^ (Figure 1). The octahedral shape was chosen based on a previous study by Dümbgen and co-workers,^27^ which indicated that the relatively large oxidation states of In^3+^ and P^3–^ constrain InP QDs to primarily assume tetrahedral or octahedral geometries. We consider the latter due to its closer resemblance to a sphere, enabling comparison with experimental findings on spherical InP QDs.^17,22^ The four structures reported in Figure 1 are derived from the defected (def) structure by removing small fragments from the QD surface, which are included in the notation. Specifically, def-InPF is obtained by removing one In, one P, and one F atom, def-F a single F atom, def-InP one In and one P atom, and def-InF_3_ one In and three F atoms. We assume that the removed atoms are in their preferred oxidation states, namely, −1 for F, + 3 for In, and −3 for P. For def-InPF and def-F, this implies that the total charge is +1 (see Figure 1). All the considered QDs include 4 3-fold coordinated phosphorus atoms on the surface, which are known to form hole traps.^27,28^ Three of the structures shown in Figure 1, namely, def-F, def-InP, and def-InF_3_, additionally embed one 3-fold coordinated In atom, which can give rise to electron traps. In the following, we denote the 3-fold coordinated In (P) surface atoms as In-3c (P-3c) (see insets in Figure 1).

**Figure 1:**
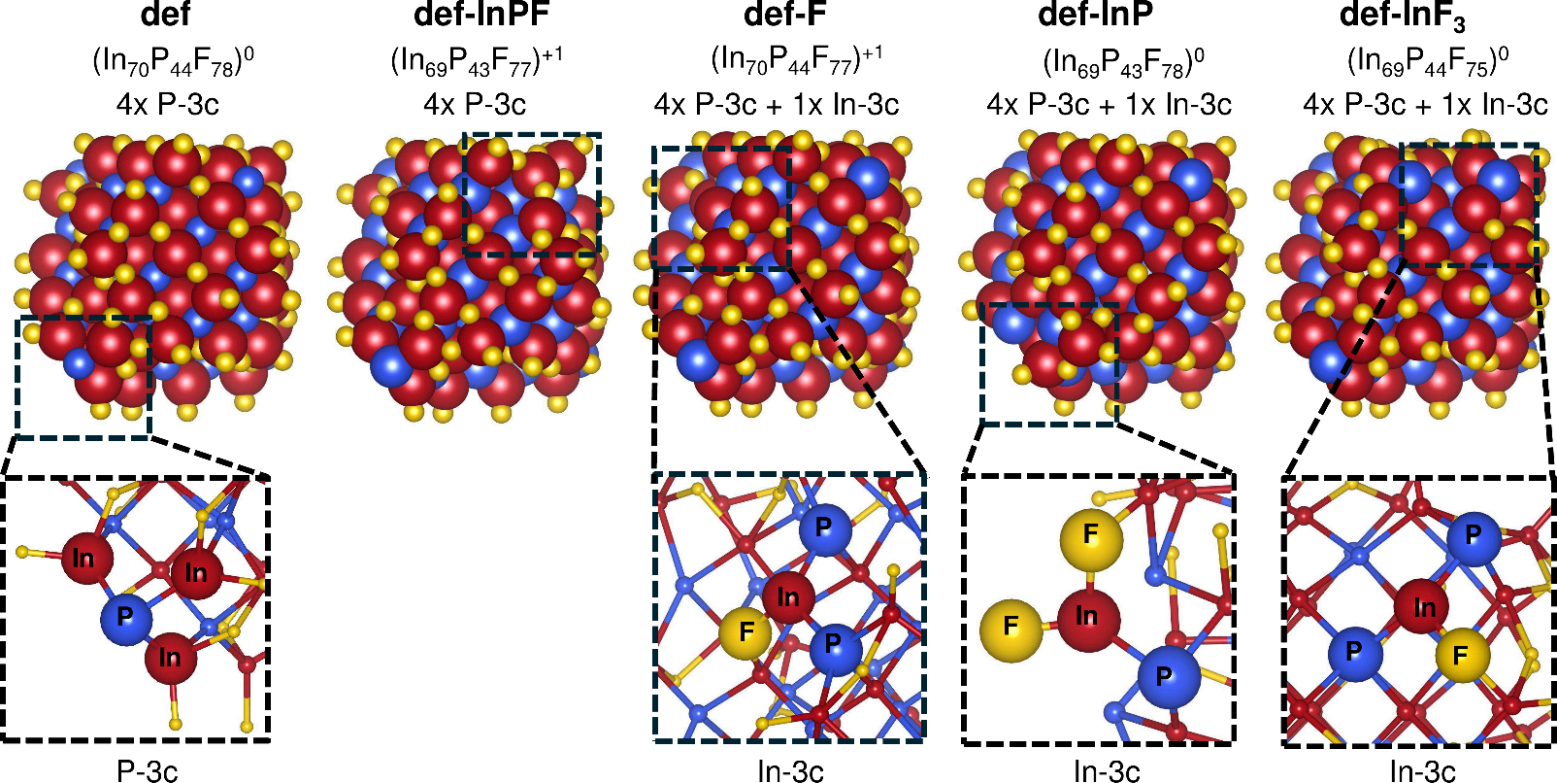
Optimized structures of the InP QDs considered in this study, including their chemical formulas, total charge, and number of defects visualized in the insets. F atoms are shown in yellow, In atoms in dark red, and P atoms in blue.

Following the approach adopted in previous work on defect excitons in CdSe QDs,^38^ we first identify the defect molecular orbitals (DMOs) among the 30 highest occupied and 30 lowest unoccupied states included in the SCIS calculations in Figure 2. The DMOs are identified based on the local Löwdin populations of In-3c and P-3c atoms (Figure S3 in Supporting Information), thus circumventing the localization of MOs suggested in ref 28. In all QDs, we find three occupied P-3c DMOs (black triangles in Figure 2) between the highest occupied molecular orbital (HOMO) and the lowest unoccupied molecular orbital (LUMO), localized over three of the four P-3c fragments. A similar localization was found in previous theoretical studies on InP QDs^25,27,28^ and is most likely a consequence of the energetic proximity of the three P-3c DMOs. The fourth P-3c defect does not contribute to the DMOs due to the stronger hybridization with the QD levels, as indicated by the shorter P–In bond lengths compared with the other three P-3c fragments (Table S1). This finding is consistent with a previous study,^27^ where some of the P-3c atoms contribute to many MOs, rather than forming a localized DMO.

**Figure 2:**
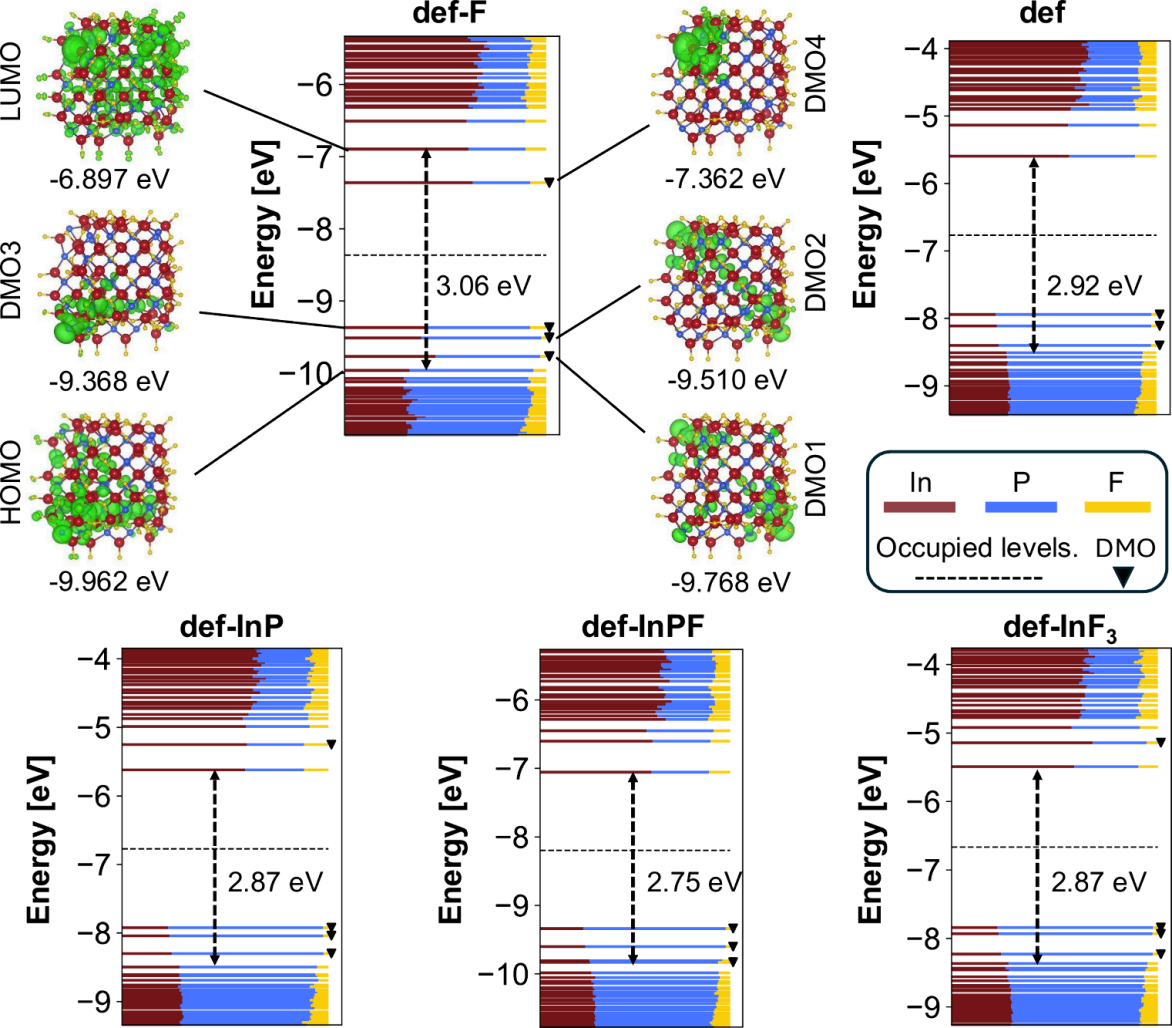
Molecular orbital energies of the highest 30 occupied and lowest 30 unoccupied states included in the SCIS calculations. The lines are colored according to the sum over the atomic Löwdin populations of different elements: In (dark red), P (blue), and F (yellow). Black dashed arrows indicate the HOMO–LUMO gaps, while the triangles mark the DMOs. The electron density plots associated with the HOMO, LUMO, and the DMOs of the def-F structure are reported along with their respective energies.

In contrast to the P-3c DMOs, the appearance of In-3c DMOS in the HOMO–LUMO gap depends on the coordination geometry of the In-3c atom, as reported previously.^28^ For def-InP and def-InF_3_ the In-3c atoms are in a trigonal planar coordination geometry and consequently the DMOs are found above the LUMO. In contrast, the In-3c defect in def-F is surrounded by 3 P^3–^ anions in a trigonal pyramidal fashion, which explains the appearance of the In-3c DMO in the HOMO–LUMO gap. Depending on the exact surface composition, we find notable changes in the HOMO–LUMO gaps, ranging from 2.75 and 3.06 eV. Further, the LUMO is delocalized over the whole QD, while the HOMO is rather delocalized on one-half of the structure, which is in agreement with previous DFT studies on defective InP QDs.^25,27,28^

To facilitate the analysis of the excitonic states, displayed in Figure 3, we categorize excitonic holes and electrons based on the contributions of specific DMOs. In our notation, holes or electrons including more than 50% DMO contribution (eqs S3 and S4) are designated as ”hole defects” (HD) or ”electron defects” (ED), respectively. Otherwise, if the DMO contribution is below 50%, holes and electrons are assumed to have ”QD” character. With this classification, we identify four types of excitons: QD-QD, HD-QD, QD-ED, and HD-ED, with the first (second) label indicating the hole (electron) character.

**Figure 3:**
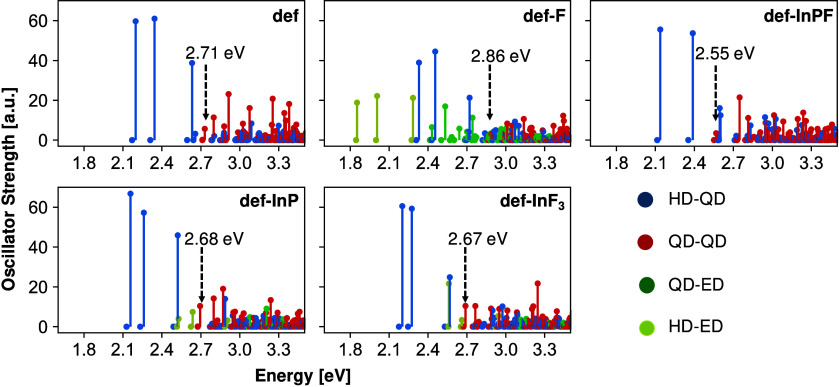
Exciton spectra calculated at the SCIS level of theory for all structures investigated in this work. The color code for the excitons refers to different types indicated in the legend, whereby QD stands for ”quantum dot”, HD for ”hole defect”, and ED for ”electron defect”. The optical gaps, corresponding to the energies of the lowest QD-QD states, are indicated by dashed arrows.

In Figure 3, the optical gaps of the QDs are marked by dashed arrows and identified as the energies of the lowest QD-QD states, representing excitons where both hole and electron have primarily QD character, corresponding to the band-edge excitons. The size of the optical gaps follows the trend of the HUMO-LUMO gaps. As observed in experiments,^16,17^ these QD-QD states tend to be relatively weak compared to the excitonic states involving in-gap defects. In particular, the HD-QD states, where the hole has defect- and the electron has QD character, are very bright across all the studied structures. Some of these HD-QD states have energies close to the band-edge QD-QD states, while others are deeper within the energy gap.

In PL experiments on quasi-spherical fluoride-passivated InP QDs,^17^ two broad PL features, energetically separated by 400 meV, are observed at 30 K. The higher peak was assigned to band-edge exciton emission, while the lower one was ascribed to emission from in-gap trap states. The energy difference between the peaks fits the relative separation between HD-QD states in the gap and the band-edge QD-QD states in all structures. For def-F, where the In-3c DMO is within the HOMO–LUMO gap, we find additional bright HD-ED and QD-ED states close to the brightest HD-QD states. In def-InF_3_, which also carries an In-3c surface defect, only very few bright HD-ED excitons and dark QD-ED states are found. This is due to the DMO being located above the LUMO in def-InF_3_, which shifts the HD-ED and QD-ED states away from the gap. In turn, this should lead to less bright HD-ED and QD-ED states compared to bright HD-QD states, which remain the major contributors to the defect PL. This result is consistent with the interpretation of previous experimental studies.^15,17^

The presence of in-gap In-3c DMOs has a dual effect on PL: it enhances defect PL while simultaneously weakening band-edge PL. In the case of def-F, the lowest QD-QD states are dark, likely due to their energetic proximity with a large number of dark HD-QD and QD-ED states. In contrast, def-InF_3_ exhibits brighter QD-QD states with fewer nearby defect states. This difference can be indirectly attributed to the DMO being within the HOMO–LUMO gap in def-F. The presence of this in-gap DMO in def-F leads to a widening of the HOMO–LUMO gap, shifting the QD-QD states to higher energies. Concurrently, the QD-ED states in def-F are shifted to lower energies compared to def-InF_3_. As a consequence, in def-F, the QD-QD and QD-ED states are found in the same energy region, increasing the number of defect excitons around the band-edge excitons. The intensity of the latter can also be quenched by the energetic proximity of bright HD-QD states, as seen in def-InPF (Figure 3). This case is particularly interesting because these nearby HD-QD bright states are expected to contribute to the PL peak that is typically attributed to emission from the band-edge excitons.

To further investigate band-edge PL for the InP QDs under study, we calculate the excitonic fine structure of the lowest eight QD-QD states shown in Figure 3 and compare our findings with an earlier experimental study on the emission from band-edge excitons in spherical InP/ZnS core–shell QDs with core sizes of approximately 2.4 nm.^22^ Despite the different QD passivations adopted here and in ref 22, namely, F atoms and a ZnS shell, respectively, we argue that the two sets of results are comparable. As for F-passivated InP QDs, experimental studies showed that defects are also present at the core–shell interface of InP/ZnS QDs.^22,40^ Consequently, the presence of symmetry-breaking effects should be observed regardless of the passivation.

As shown in Figure 4, for most of the QDs, we find similar excitonic fine structures with a 3–1–3–1 splitting, where the 3-fold degenerate states are dark and the nondegenerate states are bright. In all considered QDs except def-F, the largest splitting is obtained between the lower four states and the higher four states. In def-F, the lowest four states resemble those of all the other structures, while the higher four states are dark and energetically very close to the lower ones. This difference might be explained by the mixing of QD-QD with defect states, as previously discussed.

**Figure 4:**
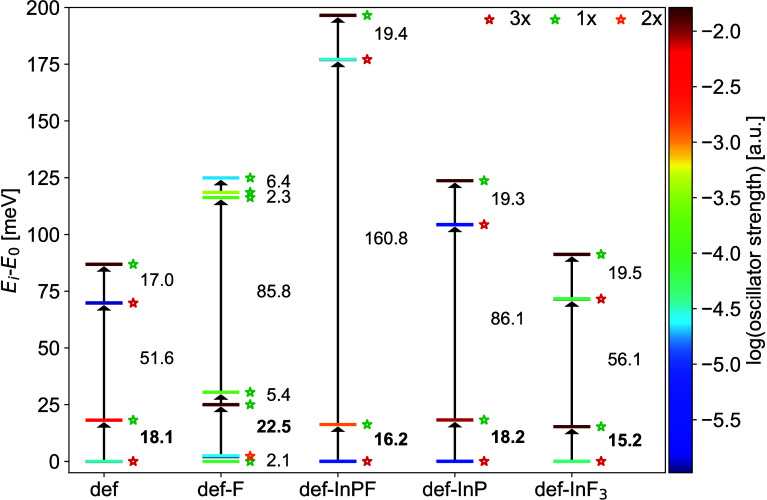
Excitonic fine structure for the lowest eight QD-QD states in the SCIS spectra of the considered InP QDs, with the lowest-energy one set to 0 meV. Each state is represented by a horizontal bar colored according to the oscillator strength shown on the sidebar in a logarithmic scale. The values reported in the plot indicate the splitting (in meV) between the excitonic states. Dark-bright splittings are highlighted in bold. Energy levels with splittings less than 0.5 meV are considered degenerate and are marked with colored asterisks: green stands for singly degeneracy, orange for double degeneracy, and red for triple degeneracy.

The dark-bright splittings between the lowest three dark states and the first bright state range between 15.2 meV in def-InF_3_ and 22.5 meV in def-F, in agreement with the dark-bright splittings of 16 meV reported for InP/ZnS QDs with core diameters of 2.4 nm.^22^ The larger splitting in our structures is expected because the exchange interactions amplifying the splittings are presumably larger in the smaller QDs. Furthermore, the PL measurements by Biadala and co-workers revealed a second, much higher-lying bright state, which also contributes to the band-edge PL.^22^ The authors argued that the emission from the second bright state is observed due to phonon bottlenecks arising from the large bright–bright splittings, which is consistent with our excitonic fine structures. In contrast, the excitonic fine structure proposed by Franceschetti et al.^29^ and originally used to explain the PL measurements, lacks a second bright state. The phonon bottleneck between the bright states may further explain the asymmetry of the band-edge PL of InP QDs in experiment,^22^ as the radiative decay from the higher-lying bright state may be faster than the nonradiative decay to the lower-lying bright state. Moreover, the sizes of the bright–bright splittings in our QDs significantly fluctuate,ranging from 68.6 meV for def to 180.2 meV for def-InPF. These large variations may explain why it was not possible to extract the bright–bright splittings from optical ensemble measurements on InP/ZnS QDs with 2.4 nm core sizes in ref 22. Further analysis revealed that the bright–bright splittings correlate with the energy differences between the HOMO and HOMO–1, see Figure S2.

While our dark-bright splittings are in good agreement with experimental values,^22^ our excitonic fine structures largely differ from the one previously used to explain the dark-bright splitting, obtained from SCIS calculations on spherical zincblende InP QDs with *T*_*d*_ symmetry.^29^ In those calculations, a 5-fold degenerate dark excitonic ground state was obtained, followed by a 3-fold degenerate bright state. This 5–3 splitting contrasts with the 3–1–3–1 splitting found here. This difference can be explained by the symmetry breaking in the single-particle wave functions (Figure 2), originating from the asymmetry of the fluoride ligand shell and the asymmetric removal of fragments from the QD surface. Both effects were ignored in the QD models used by Franceschetti et al.^29^ Additional calculations on a tetrahedral InP QD reveal the same 3–1–3–1 splitting (see Figure S3), indicating that the symmetry breaking determines the excitonic fine structures rather than the QD shapes. Thereby, for all QDs the lower four states originate from HOMO → LUMO transitions, while the higher four states are derived from HOMO–1 → LUMO excitations. Although the spin is not a good quantum number in the presence of spin–orbit coupling, the 3-fold degenerate dark states can approximately be identified as triplet states, while the bright states as singlet states.

In summary, using two-component DFT and SCIS calculations, we investigated the role of different types of excitons in the PL of quasi-spherical defective InP QDs. In agreement with previous experimental studies,^15,17^ we identified HD-QD states as the major contributors to the trap-state PL. Furthermore, in line with previous observations,^17^ we showed that In-3c electron traps are mainly responsible for band-edge PL quenching. Additionally, we found that In-3c traps can strongly contribute to the trap PL, which was never mentioned in previous studies. Importantly, to accurately determine the band-edge exciton fine structure, the consideration of realistic surfaces leading to symmetry breaking is crucial, revealing in this case a 3–1–3–1 splitting instead of a 5–3 predicted in previous work that adopted highly symmetric QDs.^29^ The dark-bright splittings extracted from our excitonic fine structures are in good agreement with previous PL experiments,^22^ validating our results. In conclusion, our study provides a comprehensive understanding of the PL properties of quasi-spherical defective InP QDs, underscoring the significant influence of surface imperfections on both trap-state and band-edge PL and providing an accurate framework for interpreting their optical properties. Our findings also play a role in a broader context, pointing out the importance of considering symmetry-breaking effects induced by surface defects and the ligand environment for a proper description of the excitonic fine structures in semiconducting quantum dots.

## Supplementary Material





## Notes

Computational methodology; inverse participation ratio plots for all structures; defect molecular orbitals identification; theory for calculating defect contributions to excitonic states; comparison of P-3c defect bond lengths with In in the “def” structure; summary of energy gaps and excitonic splittings; band-edge excitonic fine structure of a tetrahedral dot, and correlation between bright–bright exciton and HOMO–HOMO–1 splittings (PDF)
Cartesian coordinate (xyz) files for all structures under investigation (ZIP)
