# Supplementary material for: Surface Defects and Symmetry Breaking Impact on the Photoluminescence of InP Quantum Dots
Source: Nano Lett. 2025 Jun 17;25(26):10588–93. doi: 10.1021/acs.nanolett.5c02317 (PMC12232388; doi:10.1021/acs.nanolett.5c02317)
Supplement: Supplementary file 1 [file nl5c02317_si_001.pdf]

SUPPORTING INFORMATION
Surface Defects and Symmetry Breaking Impact on the Photoluminescence of InP Quantum Dots

Surender Kumar,[*,†,¶] Caterina Cocchi,[‡,¶] and Torben Steenbock[*,†]

†*Department of Chemistry, University of Hamburg, HARBOR, Building 610, Luruper Chaussee 149, Hamburg, 22761 Germany.*

‡ *Institute of Physics and Center for Nanoscale Dynamics (CeNaD), Carl von Ossietzky Universität Oldenburg, 26129 Oldenburg, Germany.*

¶*Institut für Festkörpertheorie und -Optik, Friedrich-Schiller-Universität Jena, 07743 Jena, Germany*

E-mail: surendermohinder@gmail.com,torben.steenbock@uni-hamburg.de;

Computational Methods

Geometry optimizations were performed using TURBOMOLE 7.5,[1] using the Perdew–Burke–Enzerhof (PBE) exchange-correlation functional.[2–5] In these calculations, we employed Ahlrichs' double-ζ split-valence basis set with polarization functions on all atoms (def2-SVP),[6] along with Grimme's third-generation (D3) empirical dispersion correction.[7] To accelerate the computation of Coulomb integrals in the self-consistent field (SCF) algorithm, the multipole-accelerated resolution of identity approximation (MARIJ)[6,8–10] was applied. All structures were optimized with convergence criteria of 10^{-7} Ha for the energies and 10^{-4} Ha/bohr for the gradients.

Following geometry optimization, two-component single-point calculations were conducted using the exact two-component (X2C)[11] decoupling approach to the one-electron Dirac equation in the local variant (DLU approximation).[12] These calculations employed the x2c-SVPall-2c basis set,[13] the PBE0 hybrid functional (25% exact Hartree-Fock exchange),[2–5,14] the MARIJ approximation, and the screened nuclear potential approximation for spin-orbit interaction (SNSO)[15] with modified parameters.[16,17]

Using the molecular orbitals (MOs) obtained from the two-component DFT calculations, screened configuration interaction singles (SCIS)[18] calculations were performed using an in-house developed package. These calculations included the 30 highest occupied and 30 lowest unoccupied band states, ensuring convergence of the lowest quantum dot (QD) state splittings to within 0.1 meV. The Penn model,[18–20] was utilized to describe the dielectric response function in the SCIS calculations. Additionally, the local Löwdin populations for MOs and excitonic holes and inverse participation ratio (IPR) values, were analyzed using a local version of the ARTAIOS[21] program, described in detail in previous work.[22]

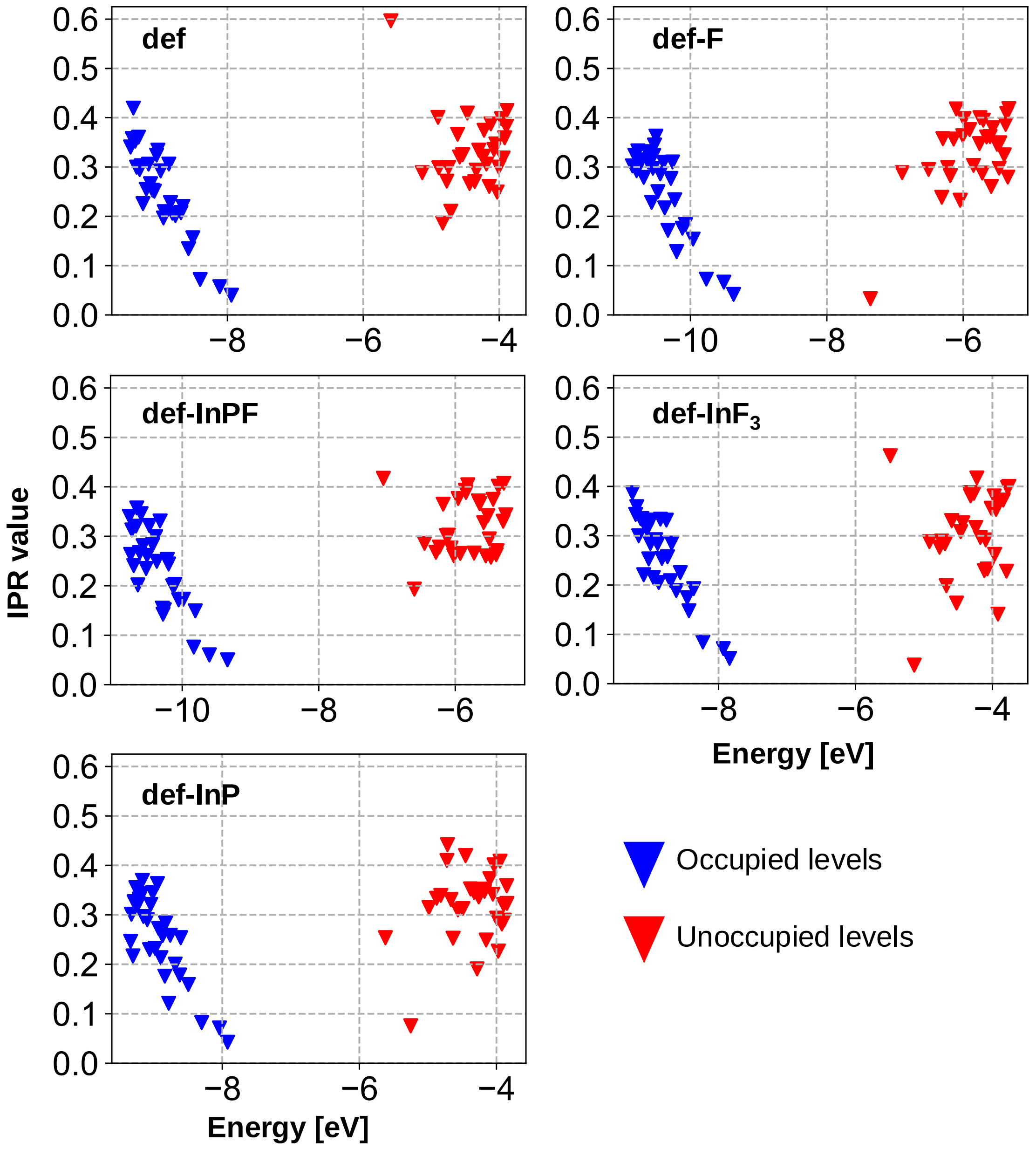

Figure S1: Inverse participation ratio (IPR) calculated from Löwdin populations[22,23] for the 30 highest occupied and 30 lowest unoccupied molecular orbitals of the investigated structures.

Inverse participation ratio plots

In Figure S1, we present the IPR plots for the 30 highest occupied and 30 lowest unoccupied MOs (see also Figure 2 of the main text) of the investigated structures. The atomic

contribution to the jth MO, assuming a Löwdin-orthogonalized basis, is defined as:

$$P_{j,A}^{\mathrm{MO}} = \sum_{\mu \in A} \left| c_{\mu j}^{\uparrow} \right|^2 + \left| c_{\mu j}^{\downarrow} \right|^2 , \tag{S1}$$

where μ refers to basis functions on atom A, and $c_{\mu j}^{\uparrow}$ and $c_{\mu j}^{\downarrow}$ are the complex MO coefficients of the two-component wave function. Using $P_{j,A}^{\mathrm{MO}}$, the IPR of the jth MO is computed as[23]

$$\mathrm{IPR}^{\mathrm{MO}} = \frac{1}{M \sum_{A}^{M} P_{j,A}^{\mathrm{MO}}}, \tag{S2}$$

where M is the total number of atoms in the system. The IPR ranges from 1 for completely delocalized MOs (equal contribution from all atoms) to $1/M$ for fully localized MOs (contribution from a single atom). All states with an IPR value below 0.1 are classified as localized.

Defect Molecular orbitals identification

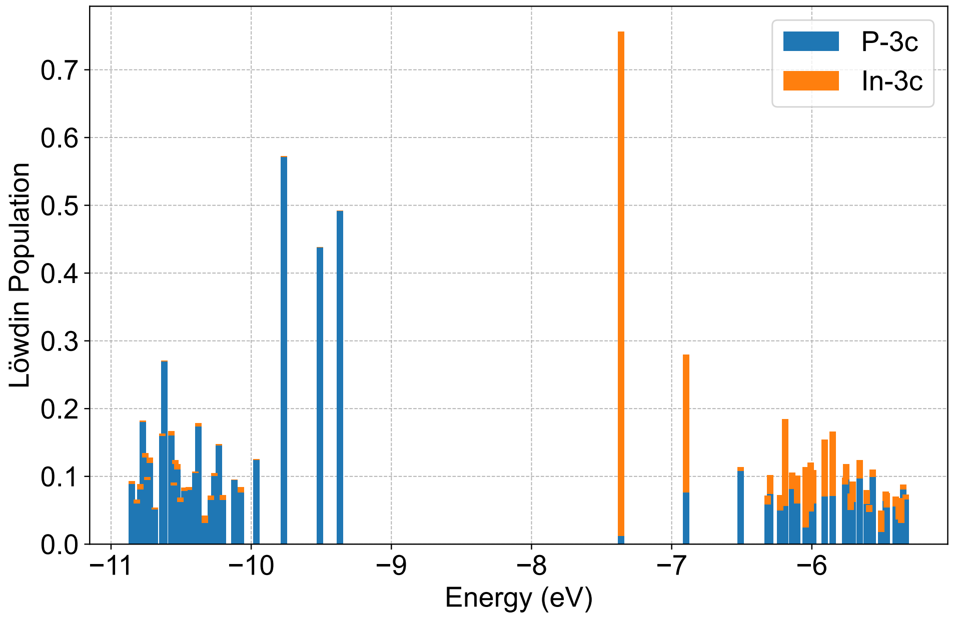

Figure S2: The Löwdin populations of the In-3c and P-3c defect atoms to the 30 highest valence states and 30 lowest conduction states for def-F structure (see main text). The values are obtained by summing up the MO populations for the spinors belonging to a Kramer's pair.

Defect contribution to the excitonic states

The absolute squared value of the SCIS coefficients C_{ia}^{N} represents the weight of a single-particle excitation from an occupied MO with index i to an unoccupied MO with index a in the Nth excitonic state. To quantify the contribution of defect-localized molecular orbitals (DMOs) to a given exciton, we define the defect contributions for hole and electron components separately. The hole defect contribution is computed as:

$$= \sum_{\substack{ia \\ i \in \mathrm{DMO}}} \left| C_{ia}^{N} \right|^2 \cdot 100\%, \tag{S3}$$

and the electron defect contribution as:

$$= \sum_{\substack{ia \\ a \in \mathrm{DMO}}} \left| C_{ia}^{N} \right|^2 \cdot 100\%. \tag{S4}$$

These values range from 0% (no DMO involvement) to 100% (solely DMO contributions), indicating the to which extent the defect states participate in the excitons.

Bond-lengths for different P-3c defect sites

Table S1: In–P bond lengths (in Å) for different P-3c defect sites in the def structure. The column labeled as "DMO" indicates whether the structural motif leads to the formation of a localized defect molecular orbital.

Defect Site	In–P Bond 1 (Å)	In–P Bond 2 (Å)	In–P Bond 3 (Å)	DMO
P-3c Defect 1	2.570	2.564	2.563	Yes
P-3c Defect 2	2.552	2.566	2.593	Yes
P-3c Defect 3	2.574	2.557	2.567	Yes
P-3c Defect 4	2.536	2.519	2.551	No

Energy Gaps and Splittings.

Table S2: Energy gaps and splittings in the considered QDs, including single-particle (SP) gaps from DFT, many-body (MB) gaps from SCIS, exciton binding energies (BE), dark-bright (DB) splittings between the lowest dark and bright states, and bright-bright (BB) splittings between the lowest bright excitonic states.

	**def**	**def-F**	**def-InPF**	**def-InP**	**def-InF$_3$**
HOMO–HOMO-1 splitting [meV]	63	111	176	113	69
SP gap [eV]	2.92	3.06	2.75	2.87	2.87
MB gap [eV]	2.71	2.86	2.55	2.68	2.67
BE [meV]	21	20	20	20	20
DB splitting [meV]	18.1	22.5	16.2	18.2	15.2
BB splitting [meV]	68.6	91.2	180.2	105.4	75.6

Band-edge excitonic fine structure of tetrahedral dot

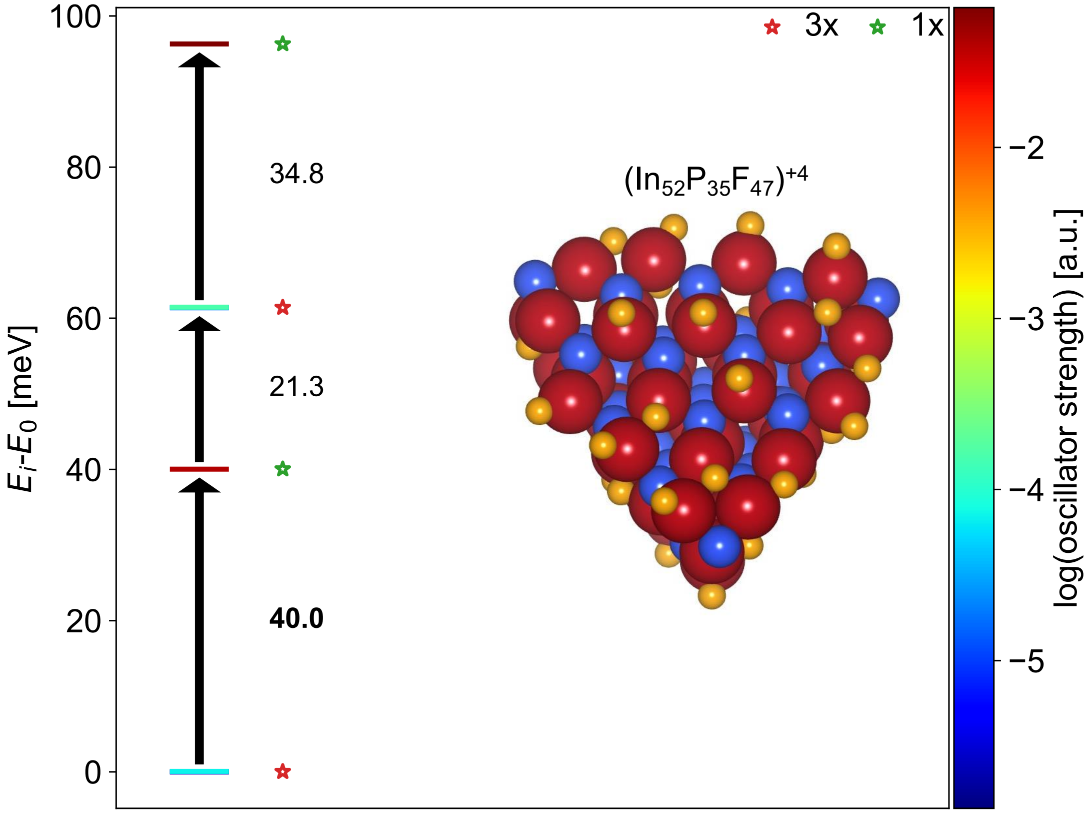

Figure S3: Excitonic fine structure including the lowest 8 band-edge excitons for a tetrahedral $(In_{52}P_{35}F_{47})^{4+}$ quantum dot, with the lowest-energy exciton state set as the reference at 0 meV. Each state is color-coded based on its oscillator strength, displayed on a logarithmic scale. Numerical labels indicate energy splittings (in meV) between excitonic levels, with dark–bright splittings emphasized in bold. Degeneracies are denoted by colored asterisks: green for non-degenerate (singly degenerate), orange for doubly degenerate, and red for triply degenerate states.

Correlation of bright-bright and HOMO–HOMO-1 splitting

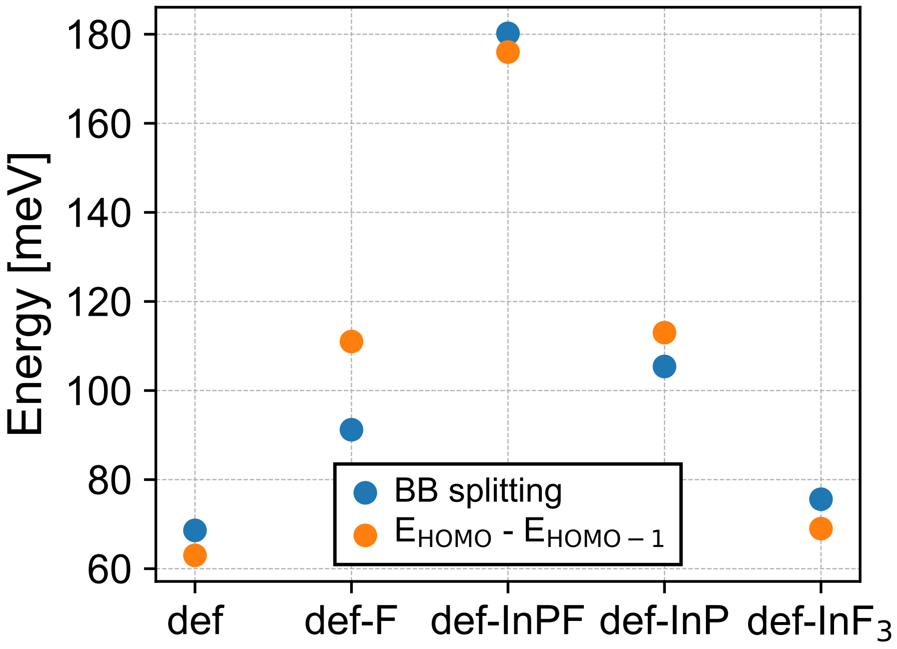

Figure S4: Correlation between bright-bright (BB) splitting of the fourth and sixth excitonic states and the single-particle HOMO–HOMO-1 energy splitting for all considered structures.

References

(1) Ahlrichs, R.; Bär, M.; Häser, M.; Horn, H.; Kölmel, C. Electronic structure calculations on workstation computers: The program system turbomole. *Chem. Phys. Lett.* **1989**, *162*, 165–169.

(2) Dirac, P. A. M. Quantum mechanics of many-electron systems. *Proc. Royal Soc. (London) A* **1929**, *123*, 714–733.

(3) Slater, J. C. A simplification of the Hartree-Fock method. *Phys. Rev.* **1951**, *81*, 385.

(4) Perdew, J. P.; Wang, Y. Accurate and simple analytic representation of the electron-gas correlation energy. *Phys. Rev. B* **1992**, *45*, 13244.

(5) Perdew, J. P.; Burke, K.; Ernzerhof, M. Generalized Gradient Approximation Made Simple. *Phys. Rev. Lett.* **1996**, *77*, 3865–3868.

(6) Weigend, F. Accurate Coulomb-fitting basis sets for H to Rn. *Phys. Chem. Chem. Phys.* **2006**, *8*, 1057–1065.

(7) Grimme, S.; Antony, J.; Ehrlich, S.; Krieg, H. A consistent and accurate ab initio parametrization of density functional dispersion correction (DFT-D) for the 94 elements H-Pu. *J. Chem. Phys.* **2010**, *132*, 154104.

(8) Eichkorn, K.; Treutler, O.; Öhm, H.; Häser, M.; Ahlrichs, R. Auxiliary basis sets to approximate Coulomb potentials. *Chem. Phys. Lett.* **1995**, *240*, 283–290.

(9) Eichkorn, K.; Weigend, F.; Treutler, O.; Ahlrichs, R. Auxiliary basis sets for main row atoms and transition metals and their use to approximate Coulomb potentials. *Theor. Chem. Acc.* **1997**, *97*, 119–124.

(10) Sierka, M.; Hogekamp, A.; Ahlrichs, R. Fast evaluation of the Coulomb potential for electron densities using multipole accelerated resolution of identity approximation. *J. Chem. Phys.* **2003**, *118*, 9136–9148.

(11) Peng, D.; Middendorf, N.; Weigend, F.; Reiher, M. An Efficient Implementation of Two-Component Relativistic Exact-Decoupling Methods for Large Molecules. *J. Chem. Phys.* **2013**, *138*, 184105.

(12) Peng, D.; Reiher, M. Local relativistic exact decoupling. *J. Chem. Phys.* **2012**, *136*, 244108.

(13) Pollak, P.; Weigend, F. Segmented contracted error-consistent basis sets of double-and triple-ζ valence quality for one-and two-component relativistic all-electron calculations. *J. Chem. Theory Comput.* **2017**, *13*, 3696–3705.

(14) Perdew, J. P.; Ernzerhof, M.; Burke, K. Rationale for Mixing Exact Exchange with Density Functional Approximations. *J. Chem. Phys.* **1996**, *105*, 9982–9985.

(15) Boettger, J. Approximate two-electron spin-orbit coupling term for density-functional-theory DFT calculations using the Douglas-Kroll-Hess transformation. *Phys. Rev. B* **2000**, *62*, 7809.

(16) Filatov, M.; Dyall, K. G. On convergence of the normalized elimination of the small component (NESC) method. *Theor. Chem. Acc.* **2007**, *117*, 333–338.

(17) Zou, W.; Filatov, M.; Cremer, D. Analytical energy gradient for the two-component normalized elimination of the small component method. *J. Chem. Phys.* **2015**, *142*, 214106.

(18) Franceschetti, A.; Fu, H.; Wang, L. W.; Zunger, A. Many-body pseudopotential theory of excitons in InP and CdSe quantum dots. *Phys. Rev. B* **1999**, *60*, 1819–1829.

(19) Kumar, S.; Bui, H.; Bester, G. Empirical band-gap correction for LDA-derived atomic effective pseudopotentials. *Comput. Condens. Matter* **2024**, *40*, e00917.

(20) Steenbock, T.; Dittmann, T.; Kumar, S.; Bester, G. Ligand-Induced Symmetry Breaking as the Origin of Multiexponential Photoluminescence Decay in CdSe Quantum Dots. *J. Phys. Chem. Lett.* **2023**, *14*, 8859–8866.

(21) Deffner, M.; Gross, L.; Steenbock, T.; Voigt, B. A.; Solomon, G. C.; Herrmann, C. ARTAIOS - a transport code for postprocessing quantum chemical electronic structure calculations, available from `https://www.chemie.uni-hamburg.de/institute/ac/arbeitsgruppen/herrmann/software/artaios.html`, Year = 2009-2017, note=(accessed 2025-06-07).

(22) Steenbock, T.; Drescher, E.; Dittmann, T.; Bester, G. How Surface Defects Shape the

Excitons and Photoluminescence of Ultrasmall CdSe Quantum Dots. *Chem. Mater.* **2024**, *36*, 6504–6514.

(23) Calixto, M.; Romera, E. Inverse participation ratio and localization in topological insulator phase transitions. *J. Stat. Mech.: Theory Exp* **2015**, *2015*, P06029.
